# The landscape of lncRNAs in gastric cancer: from molecular mechanisms to potential clinical applications

**DOI:** 10.3389/fphar.2023.1237723

**Published:** 2023-08-21

**Authors:** Jéssica Manoelli Costa Silva, Eliel Barbosa Teixeira, Ronald Matheus da Silva Mourão, Rafaella Sousa Ferraz, Fabiano Cordeiro Moreira, Paulo Pimentel de Assumpção, Danielle Queiroz Calcagno

**Affiliations:** ^1^ Núcleo de Pesquisas em Oncologia, Universidade Federal do Pará, Belem, Pará, Brazil; ^2^ Laboratório de Genética Humana e Médica, Universidade Federal do Pará, Belem, Pará, Brazil

**Keywords:** long non-coding RNAs, gastric cancer, gastric carcinogenesis, historical overview, molecular mechanisms, biomarkers

## Abstract

Gastric cancer (GC) is a highly prevalent and deadly malignant neoplasm worldwide. Currently, long non-coding RNAs (lncRNAs) have recently been identified as crucial regulators implicated in GC development and progression. Dysregulated expression of lncRNAs is commonly associated with enhanced tumor migration, invasiveness, and therapy resistance, highlighting their potential as promising targets for clinical applications. This review offers a comprehensive historical overview of lncRNAs in GC, describes the molecular mechanisms, and discusses the prospects and challenges of establishing lncRNAs as precision biomarkers.

## 1 Introduction

Gastric Cancer (GC) is a significant public health challenge due to its high incidence and mortality rates. The frequency of GC is correlated with biological sex, ethnicity, and geographic regions. In 2020, the estimated number of new cases exceeded 1 million, with approximately 768,793 associated deaths, encompassing both men and women. Table 1 provides an overview of the prevalence of GC relative to other cancer types, emphasizing its significance in the global burden of disease (Sung et al., 2021).

**TABLE 1:** The top 5 cancer types worldwide, considering estimated cases and deaths for both men and women.

Cancer site	Incidence		Mortality		PubMed
	No. of cases	% of all sites	No. of deaths	% of all sites	No. of publications^a^
Female Breast	2,261,419	11.7	684,996	6.9	3,277
Lung	2,206,771	11.4	1,796,144	18.0	4,169
Prostate	1,414,259	7.3	375,304	3.8	1,303
Colon	1,148,515	6.0	576,858	5.8	3,238
Stomach	1,089,103	5.6	768,793	7.7	2,456

^a^ Publications related to the role of lncRNAs in various types of cancer.

Source: Adapted of Sung et al., 2021.

The leading causes established for the development of GC are replication errors, environmental and hereditary factors. Among environmental factors, nutritional habits, and infections by *Helicobacter pylori* and Epstein-Barr virus stand out (Tomasetti and Vogelstein, 2015; Ashktorab et al., 2017; Tomasetti et al., 2017; Assumpção et al., 2020).

The advances in next-generation sequencing technologies have suggested that aberrant expression of non-coding RNAs (ncRNAs) plays a critical role in GC. The discovery of ncRNAs has revolutionized cancer research, opening paths for novel insights into tumor biology. Previously, ncRNAs were thought to be by-products of transcription without important biological significance. However, in the 1960s, the first speculations on the regulatory function of RNA molecules emerged, and since then, they have been identified as key players in various physiological and pathological processes.

NcRNAs can be classified into two categories based on their length: short ncRNAs and long ncRNAs (lncRNAs). Short ncRNAs in the context of GC have been extensively studied, while there is a growing interest in exploring the potential clinical applications of lncRNAs (Denaro et al., 2019; Ahmad et al., 2021).

Based on data obtained from PubMed from 2010 to 2023, 2,456 articles were published investigating the relationship between GC and lncRNAs. GC stands out among the top five cancer types frequently associated with lncRNAs, as indicated in Table 1. These studies have made significant advancements in establishing the connections between lncRNAs and essential biological processes in GC, including cell proliferation, metabolic alterations, metastasis, and therapy resistance (Cao et al., 2021; Chen Y. et al., 2021; Ding et al., 2021).

This review explores the fundamental characteristics and historical perspective of lncRNAs in GC pathogenesis. Specifically, we focus on their regulatory roles in proliferation, invasion, epithelial-mesenchymal transition, and therapeutic response. Furthermore, we address the prospects and challenges associated with the clinical implementation of lncRNAs as precision biomarkers. By thoroughly examining these aspects, we aim to provide new insights into the potential use of lncRNAs as therapeutic targets and promising biomarkers for the effective GC management.

## 2 Key biological features and mechanisms of LncRNAs

The lncRNAs represent the most abundant group of ncRNAs, comprising transcripts longer than 200 nucleotides with minimal or absent protein-coding potential (Dahariya et al., 2019; Hartford and Lal, 2020). According to the manually curated GENCODE v41 database, the total estimated number of human lncRNA genes is 19,095 (54,291 transcripts). Other lncRNA databases such as NONCODE and LNCipedia proved higher estimates. NONCODE reports 96,411 human lncRNA genes and 173,112 transcripts, while LNCipedia suggests 56,946 and 127,802 transcripts (Volders et al., 2019; Zhao et al., 2021).

Initially, the description of lncRNAs was limited to those transcribed from intergenic regions. However, it is now understood that lncRNAs can originate from various regions within the genome, including the mitochondrial genome, DNA regulatory elements, 3′and 5′untranslated regions (UTRs), and nuclear genomic loci in both sense and antisense orientations relative to protein-coding genes (Dahariya et al., 2019; Mattick et al., 2023).

Similar to messenger RNA, most lncRNAs are transcribed by RNA polymerase II (RNAPII) and undergo splicing, polyadenylation, and 5′cap addition. Furthermore, lncRNAs typically exhibit a reduced number of exons and are expressed at lower levels than coding RNAs (Mattick et al., 2023). LncRNAs can undergo diverse processing mechanisms, such as non-sequential intron splicing (back splicing) to form circular RNAs (circRNAs) or capping at both ends by small nuclear RNAs (snoRNAs) (Xing and Chen, 2018). In some instances, the lncRNAs undertake post-transcriptional cleavage, leading to the formation of a helix at the 3′end as an alternative mechanism to protect against nucleolytic cleavage (Schmitz et al., 2016; Schlackow et al., 2017; Wang et al., 2017; Dahariya et al., 2019).

The primary sequences of lncRNAs show limited conservation across different species or even within the same species, making functional characterization difficult. Proteins are commonly categorized according to conserved domains and functional mechanisms, but this does not apply to lncRNAs. An example is observed in the lncRNAs Xist and Kcnq1ot1, which both suppress gene expression in cis by recruiting the Polycomb Repressive Complex (PRC). Despite their shared mechanism, these lncRNAs display significant nucleotide sequence differences. This divergence suggests that factors beyond nucleotide sequences are crucial in controlling their regulatory activities (Fang and Fullwood, 2016; Kirk et al., 2018; Dahariya et al., 2019).

In contrast, the structural features of lncRNAs are highly conserved and considered relevant to determine their biological function. The formation of thermodynamically stable structures enables lncRNAs to interact with various biomolecules. These interactions involve RNA, DNA, and proteins, allowing lncRNAs to exert regulatory control over gene expression at multiple levels, including pre-transcriptional, transcriptional, post-transcriptional, translational, and post-translational processes. LncRNAs exert regulatory control through several molecular mechanisms, which can be categorized into distinct archetype (Zhang P. et al., 2019; Nandwani et al., 2021). Below are described some of them.

I) **Decoy** lncRNAs sequester specific regulatory factors, including transcription factors, RNA-binding proteins (RBPs), and catalytic proteins. LncRNAs acting as miRNA sponges are also included in this group.
II) **Scaffolds** lncRNAs serve as building blocks of ribonucleoprotein complexes (RNP) complexes that regulate gene expression through epigenetic and transcriptional control.
III) **Signals** lncRNAs are expressed at specific time points and subcellular regions, where they act as molecular signaling. Their role involves interacting with chromatin-modifying enzymes, such as histone methyltransferases, in order to silence target genes or block their transcription via chromatin remodeling.
IV) **Guide** lncRNAs recruit transcription factors, RNAPII, and RNPs to specific loci, with the targeting being dependent on the biological context.

Detecting lncRNAs in human circulation further enhances their potential as targets for clinical applications. Extensive research has revealed the presence of lncRNAs in body fluids, including peripheral blood, gastric juice, and saliva (Anfossi et al., 2018). Notably, Shao et al. (2016) have demonstrated that the levels of lncRNAs in plasma are unchanged for up to 8 freeze-thaw cycles under different incubation temperatures (4°C and 20°C). The stability can be explained by their packing in extracellular vesicles such as apoptotic bodies, microvesicles, and exosomes. Circulating exosomal lncRNAs have emerged as promising biomarkers for GC (Zhang et al., 2021; Badowski et al., 2022; Sun et al., 2023).

## 3 A historical perspective on the role of lncRNAs in GC

**FIGURE 1:**
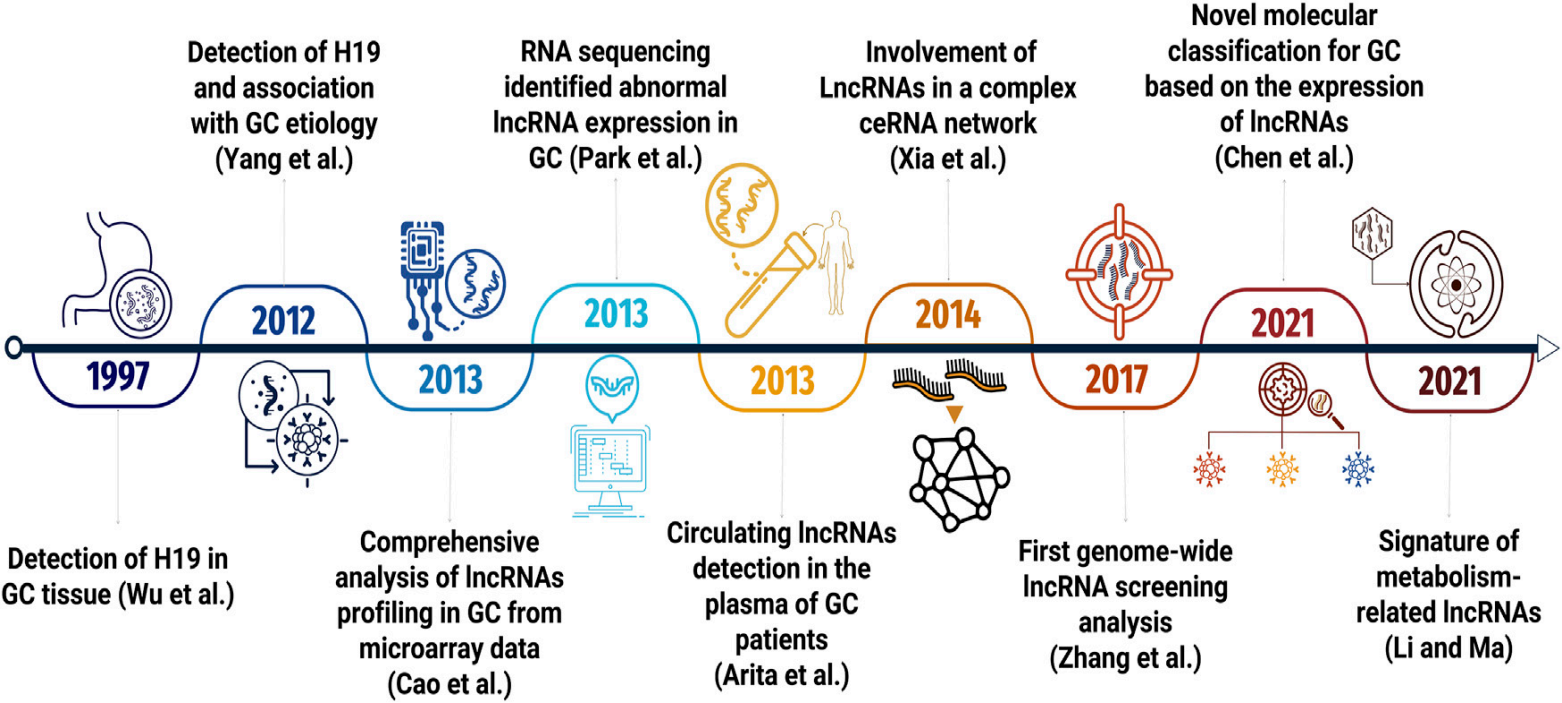
Timeline highlighting the key milestones in lncRNA research related to GC.

The investigation of lncRNAs in GC is a relatively recent field of research, originating from early studies published in the late 1990s. However, for over a decade, the scientific community primarily directed its attention towards investigating lncRNAs in other cancer types. It was not until 2012 that substantial interest emerged in unraveling the involvement of these regulatory elements in the progression of gastric tumors. Figure 1 illustrates a timeline of major GC-related lncRNAs research milestones.

The first paper to investigate the expression profiles of lncRNAs in GC was published in 1997. Wu et al. (1997) evaluated the H19 lncRNA and *IGF2* gene in a group of 70 patients diagnosed with GC, focusing on transcriptional expression, loss of imprinting, and heterozygosity. Out of the patients assessed, 28 individuals showed heterozygosity for the H19, but no significant associations with clinicopathological features were detected.

Following this study, research involving lncRNA and GC remained stagnant for a long time. It was not until 2012 that interest in investigating these regulatory elements in this type of tumor resumed. In that particular year, Yang et al. conducted a comparative analysis of H19 expression in GC tissues and adjacent tissues. They discovered that the overexpression of H19 is associated with increased cell proliferation, whereas the suppression of these lncRNA induces apoptosis in GC cell lines (Yang et al., 2012).

The first comprehensive analysis of global expression profiles of lncRNAs in GC was published in 2013. From microarray mining data in Gene Expression Omnibus, Cao et al. identified 88 differentially expressed lncRNAs between tumor and adjacent non-tumour tissue. Among the most relevant lncRNAs in the research, they found LINC00152 and PVT1. These two lncRNAs were some of the most dysregulated in the GC. Furthermore, in a validation dataset, these results were 59% similar, providing substantial evidence for the functional significance of this class of transcripts in the context of GC (Cao et al., 2013).

During the same period, parallel investigations utilizing high-throughput RNA sequencing (RNA-seq) were underway. Park et al. (2013), in a pioneering work, identified 31 intergenic lncRNAs differentially expressed in GC, findings coincident with previous work using microarray data (Park et al., 2013).

The increased levels of lncRNAs in GC tissues has prompted investigations into their presence in the bloodstream of individuals. In 2013, Arita et al. conducted a study to assess the expression of H19, HOTAIR, and MALAT1 in plasma samples obtained from GC patients and healthy controls. Only H19 showed higher levels in GC patients when compared to control, and a reduction in levels was also observed in postoperative plasma (Arita et al., 2013).

Another potential avenue in the field of investigating lncRNAs is a competing endogenous RNAs (ceRNA) hypothesis. From bioinformatics analyses, Xia et al. (2014) identified that lncRNAs may harbor microRNA response elements (MREs) and participate in a complex ceRNA network. Understanding these regulatory networks may be an alternative to developing new therapeutic approaches (Xia et al., 2014).

In the following years, extensive clinical and *in vitro* studies were conducted to elucidate the role of lncRNA in GC. Promising results showed that aberrant expression of lncRNAs is associated with the regulation of cell proliferation, invasion, apoptosis, response to treatment, tumor metastasis, and poor prognosis (Wang et al., 2014; Song et al., 2016; Zhang et al., 2017; Qin et al., 2018; Chen et al., 2020; Sun et al., 2021).

Only in 2017, the first genome-wide lncRNA screening analysis was published. The study was divided into four phases: discovery, training, validation, and external, and it brought together a total of 321 individuals. Microarray analyses revealed several differentially expressed lncRNAs; among them, five novel lncRNAs, TINCR, CCAT2, AOC4P, BANCR, and LINC00857, were detected in tumor tissue samples and pre and post-operative plasma. This signature made it possible to distinguish with high precision and sensitivity between GC patients, precancerous lesions, gastrointestinal, stromal tumors, and healthy controls. Additionally, this study demonstrated how lncRNA profiles could be highly dynamic, providing a less invasive alternative for GC monitoring and detection (Zhang et al., 2017).

Another significant advance in GC research was the development of a molecular classification based on the expression of 1,235 tumor-specific lncRNAs. Three clinically relevant molecular subtypes were identified: L1, L2, and L3 confirmed by microarray data analysis. The L3 subtype showed a worse prognosis, potentially due to the abundance of oncogenic lncRNAs, such as DUXAP8 and H19, associated with tumor progression. These results emphasize the dynamic nature of lncRNA expression and their utility as reliable prognostic markers for GC (Chen Y. et al., 2021).

More recently, lncRNAs have also been associated with GC metabolism. The metabolic profiles of individual tumors are highly heterogeneous, and the molecular action of lncRNAs strongly influences metabolic pathways. In a study published by Li and Ma (2021), a signature of 1,539 metabolism-related lncRNAs was identified, which allowed the classification of GC into two subtypes with different drug sensitivities (Li and Ma, 2021).

## 4 Exploring the role of LncRNAs in the regulation of GC development and progression

Dysregulated expression of lncRNAs is a common occurrence in cancers (Qian et al., 2020). In GC, these transcripts play a crucial role in promoting carcinogenesis through the modulation of cellular mechanisms, such as proliferation, stemness, tumor immune escape, invasion, angiogenesis, and drug resistance of tumor cells (Zhang X.-Z. et al., 2020; Gui et al., 2021; Jiang et al., 2021; Razavi et al., 2021; Sun et al., 2023).

### 4.1 Emerging role of lncRNAs in GC invasion and migration

Migration and invasion are essential mechanisms for cancer progression (Friedl and Wolf, 2003). Recent studies have shed light on the role of lncRNAs in regulating these processes by influencing cytoskeleton reorganization (Tang et al., 2018; Zhang G. et al., 2019; García-Padilla et al., 2022; Raei et al., 2022).

HOXA11-AS is a lncRNA implicated in the progression and metastasis of GC cells and tissues. It exerts its effects by modulating the miR-124-3-ITGB3 axis (You et al., 2021). ITGB3, a member of the integrin family, is positively regulated in GC and plays a critical role in focal contacts during cell migration by binding to extracellular matrix (ECM) ligands (Zhu et al., 2019).

Another lncRNA, DANCR, has been associated with cell migration and invasion in GC tissues. Its expression is positively regulated through the interaction between Enhancer Of Zeste 2 Polycomb Repressive Complex 2 Subunit (EZH2) and histone deacetylase 4 (HDAC4) (Mao et al., 2017). This interaction leads to the epigenetic suppression of lncRNA-LET transcription. EZH2 overexpression in GC cells contributes to the modulation of PTEN and Akt phosphorylation, promoting epithelial-mesenchymal transition. EZH2 also regulates the expression of metalloproteinases, such as MMP-9, associated with aggressive tumors in GC (Gan et al., 2018). Although no studies in GC demonstrate the dynamics between lncRNA-EZH2-MMP, this axis is a plausible candidate for future investigations aimed at better elucidating the migration and invasion process.

The lncRNA XIST is linked to multiple carcinogenesis aspects (Yang et al., 2021). Notably, in GC cell lines, XIST has been found to promote invasion and migration via its role as a molecular sponge for miR-337, which regulates the expression of *JAK2* (Zheng W. et al., 2020). The JAK-STAT3 signaling pathway has been previously implicated in regulating cellular motility, invasion, and migration (Teng et al., 2014). Thus, the involvement of XIST in this pathway further underscores its potential oncogenic role in GC.

Recent investigation has linked the lncRNA AK025387 to promoting cancer cell migration and invasion through the MAPK signaling pathway. This study revealed a positive correlation between AK025387 expression and the genes Raf-1, MEK2, and ERK (Sun et al., 2020). The MEK/ERK pathway has been activated in various types of tumors, including GC. Moreover, the proteins involved in the MAPK pathway contribute to regulating and activating MMPs and FAK, two essential proteins involved in focal adhesion and extracellular matrix degradation (Yang and Huang, 2015).

DSCR8, another lncRNA, promotes tumor cell progression in GC patients by acting as a miR-137 sponge and positively regulating Cdc42 expression. The reorganization of the cytoskeleton during tumor cell migration and invasion is typically dependent on Cdc42-mediated stimulation. DSCR8 is also closely associated with various clinicopathological features of GC, including tumor size, metastasis, and tumor-node-metastasis (TNM) stage (Chen Z. et al., 2021).

Moreover, LINC00152 is an onco-lncRNA overexpressed in GC tissues, particularly in patients with advanced GC, and associated with poor patient outcomes. Knockdown of LINC00152 has been shown to reduce the proliferative, migratory, and invasive capacity of cell lines and the size of the xenograft tumor by regulating the miR-193b-3p/ETS1 axis (Wang et al., 2019). These findings suggest that lncRNAs are crucial in cancer development and progression and may be potential therapeutic targets.

Functional studies conducted *in vitro* and *in vivo* have provided valuable insights into the oncogenic properties of another lncRNA, LINC00355. Specifically, LINC00355 has been identified as a promoter of crucial cancer-related processes, including proliferation, migration, and invasion, while inhibiting apoptosis in GC cells. At molecular level, LINC00355 interacts with histone deacetylase 3 (HDAC3) to suppress the transcriptional activity of tumor protein-induced nuclear protein 1 (TP53INP1), a stress-responsive protein with tumor-suppressor function. This interaction triggers the epithelial-mesenchymal transition process, which is closely associated with increased metastatic potential and disease progression in cancer (Zhao et al., 2023).

These findings underscore the critical role of lncRNAs in cancer development and progression. Further research in this field will contribute to a deeper understanding of the complex mechanisms involved in lncRNA-mediated regulation of migration and invasion, resulting in improved treatment options for GC patients.

### 4.2 LncRNAs-mediated modulation of drug response in GC

The management of GC necessitates a comprehensive multimodal approach, encompassing surgical resection, adjuvant and/or neoadjuvant chemotherapy, radiation, and targeted therapy as appropriate for specific cases. Chemotherapeutic regimens commonly incorporate a variety of pharmacological compounds, including platinum agents, taxanes, and antimetabolites (Yamashita et al., 2021). Nevertheless, the frequent development of therapy resistance poses a substantial barrier to improving survival outcomes. Recently, lncRNAs have emerged as crucial drug sensitivity and resistance mechanism regulators (Ahmad et al., 2021; Liu et al., 2022).

Platinum-based agents, including cisplatin and oxaliplatin (OXA), are classified as alkylating compounds that form bonds with DNA molecules, leading to errors in pairing DNA bases. Consequently, they prevent strand separation during DNA synthesis (Bukowski et al., 2020). According to a study by Zhang et al. (2020), high levels of MALAT1 are correlated with resistance to OXA. However, when MALAT1 was silenced, cell proliferation in resistant cell lines decreased, leading to apoptosis and increased sensitivity to OXA (Zhang Z. et al., 2020).

Similarly, the lncRNA EIF3J-DT has also been implicated in chemoresistance to OXA. Functionally, EIF3J-DT modulates the expression of *ATG14*, a gene encoding a protein essential for autophagosome assembly, through two distinct mechanisms. Firstly, it directly interacts with the mRNA of ATG14, increasing its stability and expression. Secondly, it sequesters the miRNA MIR188-3p, which prevents the degradation of *ATG14*. The EIF3J-DT-MIR188-3p-ATG14 axis has been identified as a crucial pathway involved in the activation of autophagy and chemotherapy resistance in GC cells (Menon and Dhamija, 2018; Luo et al., 2021).

Another lncRNA, PCAT-1, is overexpressed in both GC tumor tissues and cisplatin-resistant cell lines, and its increased expression has been associated with chemotherapy resistance, attributed to the epigenetic repression of the *PTEN* gene. PCAT-1 achieves this repression by recruiting EZH2 and promoting enhanced trimethylation of lysine 27 on histone 3 (H3K27me3) (Li et al., 2020). Moreover, PCAT-1 functions as a ceRNA for miR-128, thereby regulating the expression of its downstream target gene, *ZEB1* (Guo et al., 2019). These findings highlight the multifaceted regulatory roles of PCAT-1 in GC pathogenesis, encompassing epigenetic modification and ceRNA-mediated gene regulation.

The lncRNA LINC00942 has also been identified as contributing to GC cisplatin resistance. Microarray analysis revealed significant upregulation of LINC00942 in chemoresistant cells, and its knockdown resulted in increased apoptosis rates. LINC00942 localizes in the cytoplasm, allowing interactions with RBPs to modulate gene expression. Notably, LINC00942 specifically interacts with Musashi2 (MSI2), an RBP known for its tumorigenic properties and involvement in key signaling pathways like NOTCH and Ras/MAPK. By inhibiting β-Trcp-mediated degradation of MSI2, LINC00942 influences the expression of c-Myc mRNA. These results emphasize the importance of the LINC00942/MSI2/c-Myc axis in regulating chemotherapy sensitivity and its potential as a target for therapeutic intervention (Zhu et al., 2022).

Furthermore, the lncRNA UCA1 plays a role in modulating sensitivity to adriamycin in GC cells. UCA1 overexpression has been demonstrated to decrease cell apoptosis through its ability to regulate miR-27b negatively (Fang et al., 2016). These findings point to the pivotal role of lncRNAs in drug resistance mechanisms, thereby highlighting their potential as therapeutic targets for GC management.

### 4.3 The emerging role of lncRNAs in GC immune responses

The immune system possesses remarkable self-renewal and cell differentiation capabilities, crucial for developing various lymphocyte lineages, such as natural killer (NK), B, and T cells. Emerging evidence highlights the pivotal role of lncRNAs in orchestrating these intricate processes with their dynamic and cell-specific expression patterns (Chen et al., 2017; Bocchetti et al., 2021).

In the context of cancer, the dysregulation of lncRNAs has been implicated in immune evasion mechanisms, impacting patient survival (Denaro et al., 2019). For instance, studies have elucidated the influence of LncRNA HCG18 in GC-exosomal cells, which promotes the polarization of M2 macrophages through the upregulation of *KLF4* and downregulation of miR-875-3p. These molecular alterations have been associated with shorter patient survival times and increased malignancy, highlighting the prognostic value of such expression profiles as well as their potential as therapeutic targets (Gambardella et al., 2020; Piao et al., 2020; Hu et al., 2021).

Moreover, the dysregulation of lncRNAs contributes to the modulation of the tumor immune microenvironment (TIME), creating a conducive milieu for cancer growth and progression. These lncRNAs impact fundamental immune response mechanisms, such as antigen presentation, regulation of T cells, and modulation of programmed death-ligand 1 (PD-L1) (Mofed et al., 2022; Pi et al., 2021).

Exploring this field, researchers have developed and tested prognostic signatures utilizing the expression patterns of specific lncRNAs. A strong association was observed between immune infiltrating status and risk scores. Patients with higher immunophenoscores have better survival rates. This score is a measure of tumor immunogenicity. These findings propose that patients can be categorized into various prognostic groups based on their lncRNA signatures. Thus, it becomes possible to explore the development of potential immune checkpoint inhibitors (Ding et al., 2021).

Several studies have explored the role of lncRNAs in regulating the Programmed Cell Death 1 (PD-1)/PD-L1 pathway. For instance, the lncRNA SNHG15 has been found to correlate positively with *PD-L1* expression in GC cell lines. Functionally, SNHG15 acts as an endogenous competitor of miR-141, leading to increased PD-L1 expression and promoting immune resistance in GC (Dang et al., 2020).

Furthermore, the lncRNA NUTM2A-AS1 has been implicated in promoting tumorigenesis and drug resistance through its modulation of PD-L1. Acting as a ceRNA for miR-376a, NUTM2A-AS1 targets the expression of *TET1* and *HIF-1A*. Moreover, the study has shown that *TET1* interacts with HIF-1A to regulate the expression of *PD-L1*. These findings suggest that lncRNAs, through the lncRNA/miRNA/mRNA axis, play a role in immune evasion by modulating *PD-L1* expression (Wang et al., 2020).

LINC00152 has also emerged as a critical regulator in tumor cell growth by modulating the infiltration of CD8^+^ T cells. LINC00152 recruits EZH2 to the promoters of chemokines *CXCL9* and *CXCL10/CXCR3*, leading to their repression. Conversely, silencing LINC00152 promotes the expression of these chemokines, resulting in increased infiltration of CD8^+^ T cells. This influx of CD8^+^ T cells into the tumor microenvironment and the expression of CXCL9 and CXCL10 may potentiate the therapeutic effects of immune checkpoint blockade, such as anti-PD-1 therapy. Hence, LINC00152’s involvement in triggering antitumor T-cell immunity underscores as a potential target for immunotherapeutic interventions (Ou et al., 2021).

In summary, lncRNAs play intricate roles in immune regulation and tumor progression. Their dysregulation impacts immune evasion, immune cell infiltration, and modulation of crucial immune checkpoint molecules. Understanding the mechanisms underlying these interactions holds promise for developing novel therapeutic strategies targeting lncRNAs in cancer immunotherapy.

## 5 LncRNAs as potential biomarkers in GC

Biomarkers are essential indicators of specific conditions, encompassing normal biological processes, pathogenic processes, or pharmacological responses (Dancey et al., 2010). In clinical practice, protein- or peptide-based biomolecules are tumor markers. However, their sensitivity and specificity are limited, and traditional markers such as carcinoembryonic antigen (CEA) and cancer antigen 19–9 (CA19-9) have demonstrated ineffectiveness in the early detection of GC (Nakamura et al., 2019). Furthermore, despite being included in GC treatment guidelines, HER2-targeted therapies have not yielded satisfactory clinical outcomes (Hecht et al., 2016; Tabernero et al., 2018). Therefore, developing precise and reliable biomarkers is crucial for effective GC management.

Fortunately, high-throughput technologies have enabled the identification of more effective biomarkers, including lncRNAs. These molecules possess notable features such as high stability, abundance in body fluids, tissue-specific expression, versatile interactions with biomolecules, and diverse roles in gene expression regulation. Consequently, lncRNAs promise improved diagnosis, prognosis, and treatment of GC (Guimarães et al., 2018; Li et al., 2021; Liu et al., 2022; Hosseini et al., 2023).

Numerous studies have underscored the potential of lncRNAs in effectively distinguishing GC patients from healthy individuals with high sensitivity and specificity. Moreover, the clinical relevance of lncRNA expression in GC has been extensively investigated. To provide an overview of the most recent research in this field, Table 2 summarizes studies published within the last 4 years.

**TABLE 2:** LncRNAs as potential biomarkers of Gastric Cancer.

LncRNA	Expression	Potential biomarker	AUC	Sample type	Clinical implication	References
CCAT1	Up	Diagnosis and prognosis	0.89	Tissue and serum	Poor survival outcomes	Xiao et al. (2021)
TCLlnc1	Up	Diagnosis and prognosis	0.97	Tissue and plasma	Tumor distant metastasis and poor survival outcomes	Hu et al. (2022)
DRAIR	Down	Diagnosis and prognosis	0.89	Tissue and plasma	Poor survival outcomes	Jin (2021)
NR038975	Up	Diagnosis	0.71	Tissue and plasma	Advanced TNM stage	Wei et al. (2021)
lncRNA-GC1	Up	Diagnosis	0.90	Tissue and serum	Advanced TNM stage	Guo et al. (2020)
	Up	Prognosis and predictive	0.70	Plasma	Poor survival outcomes and worse response to chemotherapy	Song et al. (2022)
LINC00152/CYTOR	Up	Prognosis	—	Serum	Advanced TNM stage and poor survival outcomes	Ou et al. (2021)
PTCS3	Down	Diagnosis and prognosis	0.92	Plasma	Poor survival outcomes	Zhang et al. (2020)
LINC00355	Up	Prognosis	—	Tissue and plasma	Advanced TNM stage, distant metastasis, and poor survival outcomes	Zhao et al., 2023, Zhao et al., 2020
lnc-SLC2A12-10:1	Up	Diagnosis	0.77	Tissue and plasma	Advanced TNM stage	Zheng et al. (2020)
LINC01614	Up	Prognosis	—	Tissue	Poor survival outcomes	Chen et al. (2021)
LINC00941	Up	Prognosis	—	Tissue	Poor survival outcomes	Liu et al. (2019)
CEBPA-AS1	Up	Diagnosis and prognosis	0.82	Tissue and plasma	Tumor size, Bormann type, and TNM stage	Piao et al. (2020)
H19	Up	Diagnosis	0.85	Serum	Advanced TNM Stage	Zhou et al. (2020)
HOXA11-AS	Up	Diagnosis and prognosis	0.92	Tissue and serum	Poor survival outcomes and TNM stage	Liu et al. (2019)
B3ALT5-AS1	Up	Diagnosis and prognosis	0.81	Serum	Poor survival outcomes, LNM and TNM stage	Feng et al. (2020)
SSTR5-AS1	Up	Prognosis	—	Tissue	Poor survival outcomes and distant metastasis	Cheng et al. (2020)
MIAT	Up	Diagnosis	0.89	Serum	Poor survival outcomes and TNM stage	Xu et al. (2020)
DIRC1	Up	Diagnosis and prognosis	0.77	Tissue	Poor survival outcomes	Lin et al. (2022)
HCP5	Up	Diagnosis	0.82	Serum	Differentiation, lymph node metastasis, and nerve invasion	Qin et al. (2021)
p4516	Up	Prognosis	—	Tissue	Poor differentiation, advanced TNM stage and poor survival outcomes	Nie et al. (2019)
PANDAR, FOXD2-AS1, and SMARCC2	Up	Diagnosis	0.84	Plasma	Poor differentiation and advanced TNM	Yang et al. (2019)
FAM49B-AS, GUSBP11, and CTDHUT	Up	Diagnosis	0.82	Plasma	—	Zheng et al. (2019)

For instance, overexpression of LINC00152 has been consistently observed in GC (Mao et al., 2019; Ou et al., 2021; Li et al., 2022). In the serum and tissue, LINC00152 expression levels distinguished GC patients from healthy control and played a role as a robust prognostic indicator. Specifically, overexpression of LINC00152 exhibited a positive correlation with advanced TNM stage, lymph node metastasis, tumor invasion depth, and poorer overall survival, indicating a more aggressive disease phenotype (Ou et al., 2021).

In 2022, a newly identified lncRNA TCLlnc1 has shown higher expression levels in tissues and plasma samples from GC patients than in healthy controls. TCLlnc1 levels demonstrated significant distinguished early-stage and advanced-stage GC patients from healthy individuals, with respective area under the curve (AUC) values of 0.71 and 0.97, respectively. Furthermore, their overexpression was correlated with distant metastasis. These findings indicate that TCLlnc1 holds promise as a potential diagnostic and prognostic biomarker for GC (Hu et al., 2022).

Extracellular vesicles (EVs), secreted by viable cells, have been verified to show specific information from their cells of origin, specially lncRNAs levels. Xiao et al. (2021) observed CCAT1 levels significantly higher in the serum EVs from GC patients compared with healthy controls, patients with chronic gastritis, and atypical hyperplasia. EVs CCAT1 produced an AUC value of 0.890 with a sensitivity and specificity of 79.6% and 92.6%, respectively. These researchers determined CCAT1 as a lncRNA stable in serum EVs and a potential prognostic biomarker for GC (Xiao et al., 2021).

Recently, Zhao et al. found that LINC00355 exhibits significantly higher expression levels in exosomes derived from the plasma of GC patients than in healthy controls. Moreover, its expression is markedly elevated in GC tumor tissues compared to adjacent non-tumor tissues, with a positive correlation observed between LINC00355 expression, the depth of invasion and TNM stage. Significantly, LINC00355 overexpression is associated with poorer overall survival outcomes in GC patients. Taken togheter, these findings indicate the oncogenic role of LINC00355 in GC and its potential as a diagnostic and prognostic biomarker (Zhao et al., 2023).

A comprehensive series of multi-phase studies have highlighted the potential clinical significance of lncRNA-GC1 as a valuable biomarker for various aspects of GC. The first investigation, published in 2020, encompassed 826 participants, including 522 individuals diagnosed with GC, 85 subjects with gastric precancerous lesions, and 219 healthy donors. The findings demonstrated that elevated levels of exosomal lncRNA-GC1 exhibited accuracy in effectively distinguishing between GC patients and healthy donors, as evidenced by an AUC value of 0.903 (Guo et al., 2020).

Interestingly, lncRNA-GC1 levels were significantly higher in patients with early-stage GC, intestinal metaplasia, chronic atrophic gastritis, and positive *H. pylori* infection. This suggests that lncRNA-GC1 may serve as a reliable biomarker for early GC progression detection and monitoring. In addition, lncRNA-GC1 expression demonstrated a gradual increase in correlation with the progression of TNM stage, further supporting its potential as a prognostic indicator. An essential aspect of these studies was the simultaneous evaluation of commonly used clinical markers such as CEA, CA72-4, and CA19-9. The results indicated that lncRNA-GC1 outperformed these markers in terms of diagnostic efficiency. Notably, lncRNA-GC1 expression remained consistent after treatment with RNase and exposure to multiple freeze/thaw cycles, demonstrating its robustness and stability (Guo et al., 2020).

In 2022, a retrospective study conducted across multiple medical revealed that the levels of circulating exosomal lncRNA-GC1 could effectively distinguish patients who would benefit from fluorouracil-based adjuvant chemotherapy. GC patients with lower levels of lncRNA-GC1 exhibited better responses to chemotherapy and improved survival outcomes. The consistent results across different studies and the robustness of lncRNA-GC1 expression make it an attractive candidate for further clinical validation and potential integration into routine clinical practice (Song et al., 2022).

In addition to their potential as biomarkers, lncRNAs hold promise as novel therapeutic targets. The diverse and intricate functional roles of lncRNAs provide opportunities for various therapeutic interventions. These include the modulation of lncRNA genomic loci to induce transcriptional repression, hindrance of secondary structure formation to prevent interactions with biomolecules, the introduction of synthetic lncRNAs, and modifications of expression patterns. Despite the therapeutic potential of lncRNAs, it is essential to note that no lncRNA-based therapies have yet progressed to phase II or III clinical development (Winkle et al., 2021).

The findings presented in this study highlight the promising potential of lncRNAs as valuable tools in clinical practice. However, it is crucial to acknowledge that the translation of lncRNAs into clinical applications is still in its early stages, with limited success thus far. Currently, only one lncRNA, PCA3, has been successfully translated into an FDA-approved molecular diagnostic test, namely, PCA3 ProgensaTM (Gen-Probe Inc., San Diego, CA, USA), which is primarily recommended for patients who have previously had a negative biopsy for prostate cancer (Cui et al., 2016).

### 5.1 Challenges of incorporating lncRNAs into clinical practice

Several vital aspects must be addressed to overcome the challenges of implementing lncRNAs in clinical practice. A primary challenge in lncRNA research is the limited sample size often encountered in studies. Many investigations have a relatively small number of participants, which can compromise the statistical power and precision of the results. Additionally, including healthy individuals as controls is crucial for validating the specificity and sensibility of lncRNA biomarkers. Some studies’ absence of appropriate control groups can introduce biases and limit the accurate evaluation of biomarker efficacy (Zheng, 2018).

Another significant challenge is the prevailing focus on specific regions or ethnicities in lncRNA research. This geographic bias may hinder the generalizability and reproducibility of findings in broader populations. To ensure the clinical relevance and applicability of lncRNA biomarkers, including diverse populations and considering the potential influence of genetic and environmental factors is imperative (Zheng, 2018).

The lack of standardization in pre-analytical and experimental procedures represents another challenge in the field. The absence of well-established protocols for sample collection, processing, and analysis may impede the comparability and reliability of results across different studies. The harmonization and standardization of these procedures are critical to facilitate robust comparisons between studies and enhance the overall quality (Anfossi et al., 2018).

Furthermore, retrospective study designs are prevalent in lncRNA research, which can introduce inherent biases. Prospective studies are essential to validate lncRNA biomarkers’ predictive and prognostic value. Long-term follow-up is necessary to assess the performance of these biomarkers in predicting treatment response, disease progression, and patient outcomes, thereby providing valuable insights for clinical decision-making (Anfossi et al., 2018).

In addition to technical challenges, the field of lncRNA research faces inherent obstacles related to the nature of lncRNAs. For example, the poor conservation of lncRNAs across different species poses difficulties in evaluating their functions and effects in animal models. The lack of conservation hinders the translation of findings from model organisms to humans and limits our understanding of the broader biological implications of lncRNAs (Winkle et al., 2021).

Moreover, lncRNAs are often expressed at low levels, which presents challenges in their accurate measurement and detection (Mattick et al., 2023). The quantification of lncRNAs requires sensitive and specific techniques that can reliably distinguish them from background noise and accurately determine their expression levels. Detecting specific lncRNAs in physiological processes can be challenging due to their transient or cell-type-specific expression patterns. To fully harness the potential of lncRNAs in clinical applications, further research efforts are needed to unravel their functional significance (Anfossi et al., 2018; Fathi Dizaji, 2020; Statello et al., 2021; Winkle et al., 2021).

Understanding the precise roles of lncRNAs in gene regulation, cellular processes, and GC pathogenesis is crucial for developing targeted interventions. GC can be anatomically classified into two main subtypes, cardial and non-cardial GC, each with distinct epidemiological profiles and mechanisms of carcinogenesis. However, currently, there are no available studies characterizing the expression patterns of lncRNAs based on the anatomical subtypes. Performing an exploratory investigation to identify specific lncRNA expression patterns in these subtypes is crucial for implementing a more effective screening strategy (Cao et al., 2013; Thrift et al., 2023).

## 6 Discussion

GC poses a significant public health challenge due to its high incidence and mortality rates. Therefore, the identification of precise biomarkers and novel therapeutic targets is crucial for improved management. Abnormal expression of lncRNAs plays a crucial role in the development and progression of GC. By acting as master regulators of gene expression, lncRNAs exert substantial influence on cancer hallmarks, including cell proliferation, evasion of cell death, immune evasion, and metabolic alterations.

The discovery of lncRNAs has revolutionized the field of molecular biology since the publication of the first paper in 1997. Substantial scientific progress has been made in elucidating the involvement of lncRNAs in gastric carcinogenesis.

Current studies contribute to a more comprehensive understanding of the mechanisms by which lncRNAs exert their actions in GC. LncRNAs interact with biomolecules, acting as miRNA sponges, interacting with RNA-binding proteins, and modulating the expression of critical genes within pro-tumorigenic pathways.

Notably, most lncRNAs’ expression is highly specific to tissues and cell types. Moreover, the widespread and stable presence of lncRNAs in body fluids, including blood, saliva, urine, and gastric juice, makes them promising candidates for clinical applications, including diagnostic biomarkers, prognostic indicators, predictors of therapeutic response, and potential targets for the development of personalized cancer treatment strategies.

Extensive research is currently dedicated to developing therapeutic strategies targeting lncRNAs. Multiple approaches are being explored, including antisense oligonucleotides (ASO), CRISPR/Cas9 technology, RNA interference (RNAi) using viral vectors, and nanotechnology-based delivery systems.

ASOs are single-stranded deoxyribonucleotides with complementary sequences to RNA targets. In the context of lncRNAs, the ASOs can bind to the desired lncRNA and induce degradation. Remarkably, ASOs targeting natural antisense transcripts (NATs) have demonstrated promising preclinical results in gene reactivation within the central nervous system. Similar to this approach, RNAi technology utilizes small interfering RNAs (siRNAs) or short hairpin RNAs (shRNAs) to target and silence specific lncRNAs. Viral vectors, such as lentiviruses or adenoviruses, can deliver these siRNAs or shRNAs into cells, enabling efficient knockdown of the target lncRNAs. Both ASOs and siRNAs can enhance their delivery to specific cells or tissues through nanotechnology-based systems. These nanocarriers can improve therapeutic molecules’ stability, bioavailability, and cellular uptake and enhance the efficacy of therapeutic targets. Alternatively, the CRISPR/Cas9 technology inhibits or alters the expression of lncRNAs by introducing specific modifications to the DNA sequences that transcribe lncRNAs.

Currently, no lncRNA-targeted therapeutic intervention has progressed to clinical development. Nevertheless, lncRNAs are actively investigated as potential biomarkers. The FDA has approved the first lncRNA-based diagnostic test, PCA3 ProgensaTM, for prostate cancer. Moreover, ongoing research endeavors explore the clinical relevance of lncRNAs in a diverse array of complex diseases, extending beyond cancer to neurological conditions.

Despite the potential of lncRNAs as valuable tools in GC management, much remains to be explored and understood. Challenges such as small sample sizes, incorporating adequate healthy controls, mitigating geographic bias, establishing standardized protocols, tolerability issues, inefficient intracellular delivery, and overcoming the reliance on retrospective study designs must be addressed for successful translation into routine diagnostic tests and to ensure the safety and efficacy of therapeutic interventions in clinical settings.

Continued research, integration of multi-omics approaches, a multidisciplinary team, and large-scale multicenter studies are essential to advance our understanding of lncRNAs’ role in tumorigenesis. This comprehensive approach may establish lncRNAs as robust biomarkers, thus propelling personalized management of GC.
